# Imaging of Claudin-4 in Pancreatic Ductal Adenocarcinoma Using a Radiolabelled Anti-Claudin-4 Monoclonal Antibody

**DOI:** 10.1007/s11307-017-1112-8

**Published:** 2017-08-25

**Authors:** Julia Baguña Torres, James C. Knight, Michael J. Mosley, Veerle Kersemans, Sofia Koustoulidou, Danny Allen, Paul Kinchesh, Sean Smart, Bart Cornelissen

**Affiliations:** CR-UK/MRC Oxford Institute for Radiation Oncology, Department of Oncology, University of Oxford, Old Road Campus Research Building, Off Roosevelt Drive, Oxford, OX3 7DQ UK

**Keywords:** Claudin-4, Pancreatic ductal adenocarcinoma, SPECT, Molecular imaging

## Abstract

**Purpose:**

Despite its widespread use, the positron emission tomography (PET) radiotracer 2-deoxy-2-[^18^F]fluoro-D-glucose ([^18^F]FDG) has been shown in clinical settings to be ineffective for improving early diagnosis of pancreatic ductal adenocarcinoma (PDAC). A promising biomarker for PDAC detection is the tight junction protein claudin-4. The purpose of this study was to evaluate a new single-photon emission computed tomography (SPECT) imaging agent, [^111^In]anti-claudin-4 mAb, with regard to its ability to allow visualisation of claudin-4 in a xenograft and a genetically engineered mouse model of PDAC.

**Procedures:**

The ability of [^111^In]anti-claudin-4 mAb to selectively target claudin-4 was assessed using two human xenograft tumour models with differential claudin-4 status in mice. [^111^In]anti-claudin-4 mAb was also used to detect PDAC development in genetically engineered KPC mice. The PDAC status of these mice was confirmed with [^18^F]FDG-PET, magnetic resonance imaging (MRI), histology, and immunofluorescence microscopy.

**Results:**

High uptake of [^111^In]anti-claudin-4 mAb was observed in PDAC xenografts in mice, reaching 16.9 ± 4.5 % of injected dose per gram (% ID/g) at 72 h post-injection. This uptake was mediated specifically by the expression of claudin-4. Uptake of [^111^In]anti-claudin-4 mAb also enabled clear visualisation of spontaneous PDAC formation in KPC mice.

**Conclusions:**

[^111^In]anti-claudin-4 mAb allows non-invasive detection of claudin-4 upregulation during development of PDAC and could potentially be used to aid in the early detection and characterisation of this malignancy.

**Electronic supplementary material:**

The online version of this article (doi:10.1007/s11307-017-1112-8) contains supplementary material, which is available to authorized users.

## Introduction

Pancreatic ductal adenocarcinoma (PDAC) is one of the most lethal cancer types as it has an extremely poor 5-year survival rate of < 5 % [1]. This dismal prognosis is due in part to the asymptomatic progression of this malignancy in its early stages and the lack of adequate screening measures, resulting in 80–90 % of patients being diagnosed when the disease is already in an advanced, metastatic state. The ability to identify PDAC early in its development has been shown to improve outcome, particularly if the patient can be diagnosed while still eligible for potentially curative surgical resection [2]. At present, the most commonly utilised imaging modality for suspected PDAC is x-ray computed tomography (CT); however, it is now increasingly being used in conjunction with positron emission tomography (PET) to aid in early diagnosis and staging of this malignancy [3, 4]. The PET radiotracer 2-deoxy-2-[^18^F]fluoro-D-glucose ([^18^F]FDG) is the standard clinical option for suspected PDAC; however, it has been shown to be largely ineffective for the detection of small (≤ 20 mm) pancreatic tumours, and in most cases inferior to conventional CT and magnetic resonance imaging (MRI) for the detection of liver, peritoneal and lung metastases. [^18^F]FDG is also unable to distinguish focal mass-forming pancreatitis from pancreatic cancer in most cases [5]. These limitations strongly indicate the need for alternative biomarkers which arise during the early stages of PDAC formation that can be measured noninvasively by molecular imaging techniques.

Several gene expression analyses have shown that the transmembrane protein claudin-4 is upregulated increasingly throughout PDAC formation, including in the pre-invasive pancreatic intraepithelial neoplastic (PanIn) lesions which form before PDAC is established [6–10]. While this protein is also present in healthy bladder, breast, prostate and gastrointestinal mucosa, its expression levels in these environments are very low in comparison to those in pancreatic cancer tissues [11]. Claudin-4 therefore represents an attractive biomarker for the early detection of PDAC. The ability to detect claudin-4 upregulation may provide valuable diagnostic and staging information which would supplement conventional imaging procedures and impact on the clinical decision-making process.

In pursuit of a claudin-4 imaging agent, we and others have previously exploited the favourable binding characteristics of the bacterial ligand *Clostridium perfringens* enterotoxin (CPE). In 2013, Neesse et al. modified a C-terminal fragment of CPE (cCPE) with the fluorophore Cy5.5 (Cy5.5-GST-cCPE) and showed elevated uptake of this imaging agent in PanIn lesions and PDAC compared to normal pancreases in genetically engineered mouse models of pancreatic cancer [12]. As clinical applications of fluorescence imaging are limited due to considerable signal attenuation by tissue, we subsequently developed a cCPE derivative modified with the single-photon emission computed tomography (SPECT) radioisotope indium-111 ([^111^In]cCPE-GST) [13]. Despite exhibiting a low binding affinity (1.93 ± 0.59 μM), this radiotracer revealed claudin-4-mediated tumour uptake in a variety of human cancer xenograft and genetically engineered models which were all found to have upregulated claudin-4 expression. However, overall tumour uptake was generally low, and we have since sought to investigate alternative claudin-4 targeting vectors with improved target affinity and specificity.

Radiolabelled antibodies, given their superior affinity and selectivity, have been used extensively as vectors for PET and SPECT imaging [14]. Here, we report the preclinical evaluation of an ^111^In-labelled anti-claudin-4 monoclonal antibody with the aim of providing a new clinical tool for improving upon early detection of PDAC. As *in vivo* simulations of PDAC, we have used human pancreatic duct epithelioid carcinoma xenografts in mice, and also a well-validated, clinically relevant genetically engineered model of PDAC (KPC model) [15] that develops a spectrum of premalignant PanIN lesions which ultimately progress to PDAC.

## Materials and Methods

### Materials

All reagents were purchased from Sigma-Aldrich unless otherwise stated and were used without further purification. The chelating agent *p*-SCN-Bn-DTPA was purchased from Macrocyclics Inc. (Dallas, TX). Water was deionised using a Barnstead NANOpure purification system (Thermo Scientific) and had a resistance of > 18.2 MΩ cm^−1^ at 25 °C. Protein concentration measurements were made on a ND-1000 spectrophotometer (NanoDrop Technologies, Inc.). Instant thin-layer chromatography (iTLC) was performed on glass microfiber chromatography paper (Agilent Technologies), and strips were analysed with either a Bioscan AR-2000 radio-TLC scanner (Eckert & Ziegler) or a Cyclone Plus Phosphor Imager (PerkinElmer). Radioactivity measurements were determined using a CRC®-25R dose calibrator (Capintec, Inc.).

### Antibodies

The human MAB4219 antibody (R&D Systems) was used as a targeting moiety for *in vivo* imaging of claudin-4 due to its ability to recognise an epitope in the first extracellular loop of the protein (aa Met29-Arg81). The reactivity of this antibody for both murine and human claudin-4 was confirmed by flow cytometry in claudin-4-expressing human Panc-1 and murine 4T-1 cells (see Suppl. S1).

The status of claudin-4 expression in cells and tissue was assessed by Western immunoblot and immunofluorescence (see supplementary information for full experimental protocols) using anti-claudin-4 antibodies 329400 and PA5–28830 (ThermoFisher Scientific), respectively, since MAB4219 does not perform in these techniques.

### Radiolabelling

Modification of anti-claudin-4 mAb with *p*-SCN-Bn-DTPA and subsequent radiolabelling with indium-111 were conducted following methods described by Brom et al. [16]. In brief, to a solution of anti-claudin-4 MAB4219 (200 μg) or mouse IgG_2A_ (200 μg, MAB003, R&D Systems) in 0.1 M NaHCO_3_ (pH 9, 100 μl, Chelex treated) was added 20 M equivalents of *p*-SCN-Bn-DTPA (245.6 μM) in anhydrous dimethyl sulphoxide. The reaction mixture was incubated at room temperature for 30 min with gentle shaking (450 rpm), and the excess *p*-SCN-Bn-DTPA was removed by Sephadex-G50 size exclusion chromatography (Sigma-Aldrich). The affinity of the DTPA-conjugated MAB4219 for claudin-4 was evaluated by flow cytometry in Panc-1 and HT1080 cells and compared to that of the unmodified antibody.

Indium-111 in 0.02 M hydrochloric acid (sourced from Mallinckrodt Pharmaceuticals) was added to a 2-mg/ml solution of the DTPA-modified antibody to achieve a ratio of at least 1 MBq to 1 μg. The reaction mixtures were incubated at room temperature for 1 h, and the radiolabelling efficiency was determined by iTLC using an eluent of 0.1 M sodium citrate buffer (pH 5.5). The crude reaction mixture was purified by Sephadex-G50 size exclusion chromatography, eluting with 100-μl fractions of phosphate-buffered saline (pH 7.4).

### *In Vivo*

All animal procedures were performed in accordance with the UK Animals (Scientific Procedures) Act 1986 and with local ethical committee approval. Xenograft tumours were established in the right hind flank of female athymic BALB/c *nu/nu* mice (Harlan) by subcutaneous injection of Panc-1 (1 × 10^6^) or HT1080 (1 × 10^6^) cells in DMEM (100 μl). When tumours reached a diameter of approximately 10 mm, [^111^In]anti-claudin-4 or [^111^In]mIgG (5 MBq, 5 μg) in sterile PBS (100 μl) were injected intravenously *via* the lateral tail vein (*n* = 5 for [^111^In]anti-claudin-4 and *n* = 3 for [^111^In]mIgG). SPECT/CT images were acquired using a nanoSPECT-CT scanner at 24, 48, and 72 h after injection. After the final imaging session, mice were euthanized by cervical dislocation, and selected organs, tissues and blood were removed. The amount of radioactivity in each organ was measured using a 1470 WIZARD gamma counter (PerkinElmer). Counts per minute were converted into MBq using a calibration curve generated from known standards. These values were decay-corrected to the time of injection, and the percentage of the injected dose per gram (% ID/g) of each sample was calculated.

Genetically engineered KPC mice (K-ras^LSL.G12D/+^; p53^R172H/+^; PdxCre; male, 3–4 months old), which were found to be PDAC-positive by [^18^F]FDG-PET and MRI screening, were also imaged by SPECT 72 h after [^111^In]anti-claudin-4 or [^111^In]mIgG (5 MBq, 5 μg) administration (*n* = 3 per tracer). Further details on the imaging protocols are included in the supplementary information.

### *Ex Vivo*

After imaging, pancreatic cancer tissue from athymic BALB/c nu/nu mice and KPC mice was flash-frozen with dry ice and stored at − 80 °C overnight. Frozen tissue was sectioned (8 μm) using an OTF5000 cryotome (Bright Instruments Ltd). Tissue sections were thaw-mounted onto Superfrost PLUS glass microscope slides (Menzel-Glaser, Thermo Scientific) and allowed to dry at room temperature. The slides were then exposed to a storage phosphor screen (PerkinElmer, Super Resolution, 12.5 × 25.2 cm) in a standard X-ray cassette for 15 h. The phosphor screen was then imaged using a Cyclone® Plus Storage Phosphor System (PerkinElmer), and images were analysed with OptiQuant 5.0 (PerkinElmer) and ImageJ (NIH).

After autoradiography, claudin-4 levels and PDAC morphology in *ex vivo* tissue were characterised by immunofluorescence and haematoxylin and eosin (H&E) staining, respectively. Full experimental details for H&E staining are reported in the supplementary information.

### Statistical Analyses

All statistical analyses and nonlinear regression were performed using GraphPad Prism (GraphPad Software). An extra sum-of-squares *F* test was used to compare equilibrium dissociation constants. One- or two-way ANOVA was used for multiple comparisons, with Tukey post-tests to calculate significance of differences between groups. All data were obtained in at least triplicate and results reported and graphed as mean ± standard deviation, unless stated otherwise.

## Results

### Target Validation

**Fig. 1.**
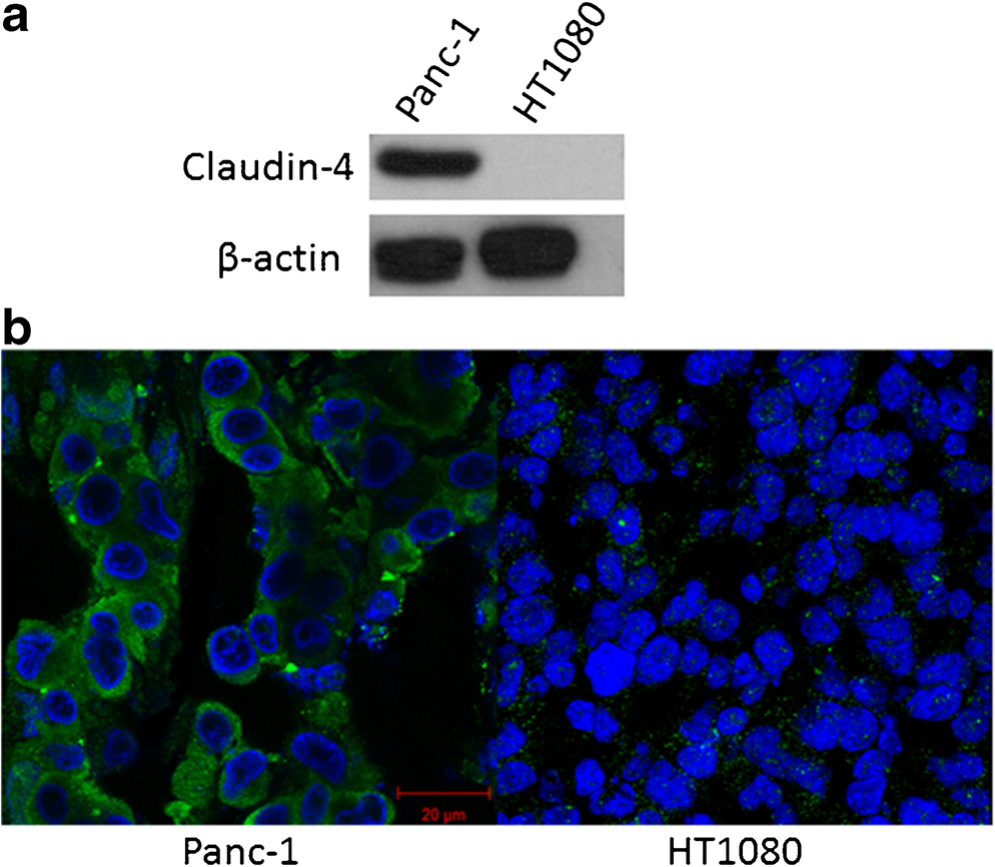
**a** Western blot confirming the presence and absence of claudin-4 on Panc-1 and HT1080 cell lysates, respectively. **b** Confocal images of immunofluorescence staining of claudin-4 (green) and nucleus (blue) in Panc-1 and HT1080 xenograft tissue. Photomicrographs show high expression of claudin-4 in Panc-1 xenografts and negligible fluorescent signal in claudin-4 negative tumour tissue.

Western blot analysis of whole cell lysates (Fig. 1a) confirmed the expression of claudin-4 in the Panc-1 cell line. In contrast, claudin-4 could not be detected by Western blot in HT1080 cells. Immunofluorescence microscopy experiments on tissue sections obtained from Panc-1 or HT1080 xenograft tumours showed the differential claudin-4 status in these two cell lines that was maintained when transferred to an *in vivo* setting (Fig. 1b)*.*

### Binding Affinity of DTPA-Anti-Claudin-4 mAb

**Fig. 2.**
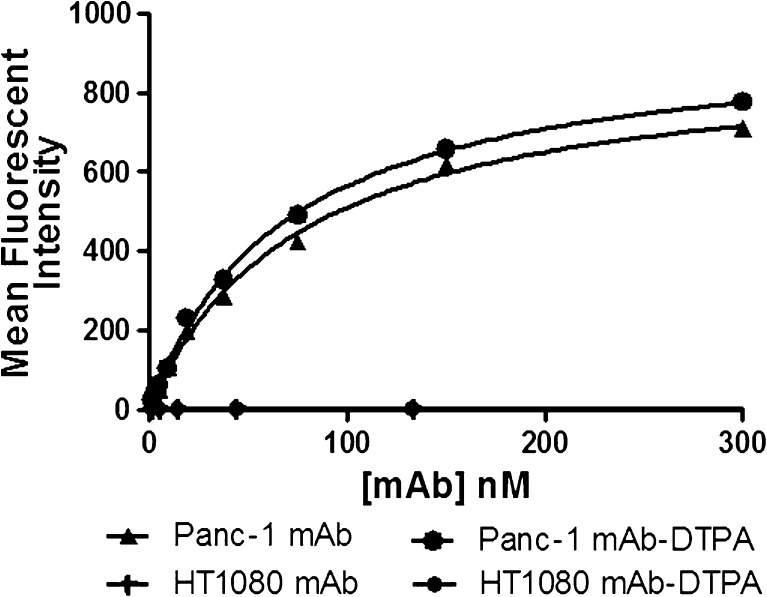
Determination of antibody affinity by flow cytometry. The binding of DTPA-anti-claudin-4 mAb to claudin-4 was found to be comparable to that of the unmodified antibody in Panc-1 cells. Both DTPA-modified and unmodified anti-claudin-4 mAbs exhibited negligible binding to the target antigen in claudin-4 negative HT1080 cells.

The binding affinity of DTPA-modified anti-claudin-4 mAb (MAB4219) (*K*_d_ = 77.5 ± 6.8 nM; Fig. 2) was shown on claudin-4 expressing Panc-1 cells to be comparable to that of the unmodified antibody (*K*_d_ = 66.3 ± 7.8 nM; *P* > 0.05; Fig. 2). Both DTPA-modified and unmodified anti-claudin-4 mAb exhibited negligible binding to claudin-4 negative HT1080 cells.

### Indium-111 Labelling of DTPA-Anti-Claudin-4 mAb

[^111^In]anti-claudin-4 mAb was routinely synthesised in excellent radiochemical yields (99.8 ± 0.2 %) and obtained in high purity (> 99 %) following G50 size-exclusion chromatography, with a typical specific activity of 1 MBq/μg.

### Imaging of Claudin-4 in Pancreatic Xenograft Tumours

**Fig. 3.**
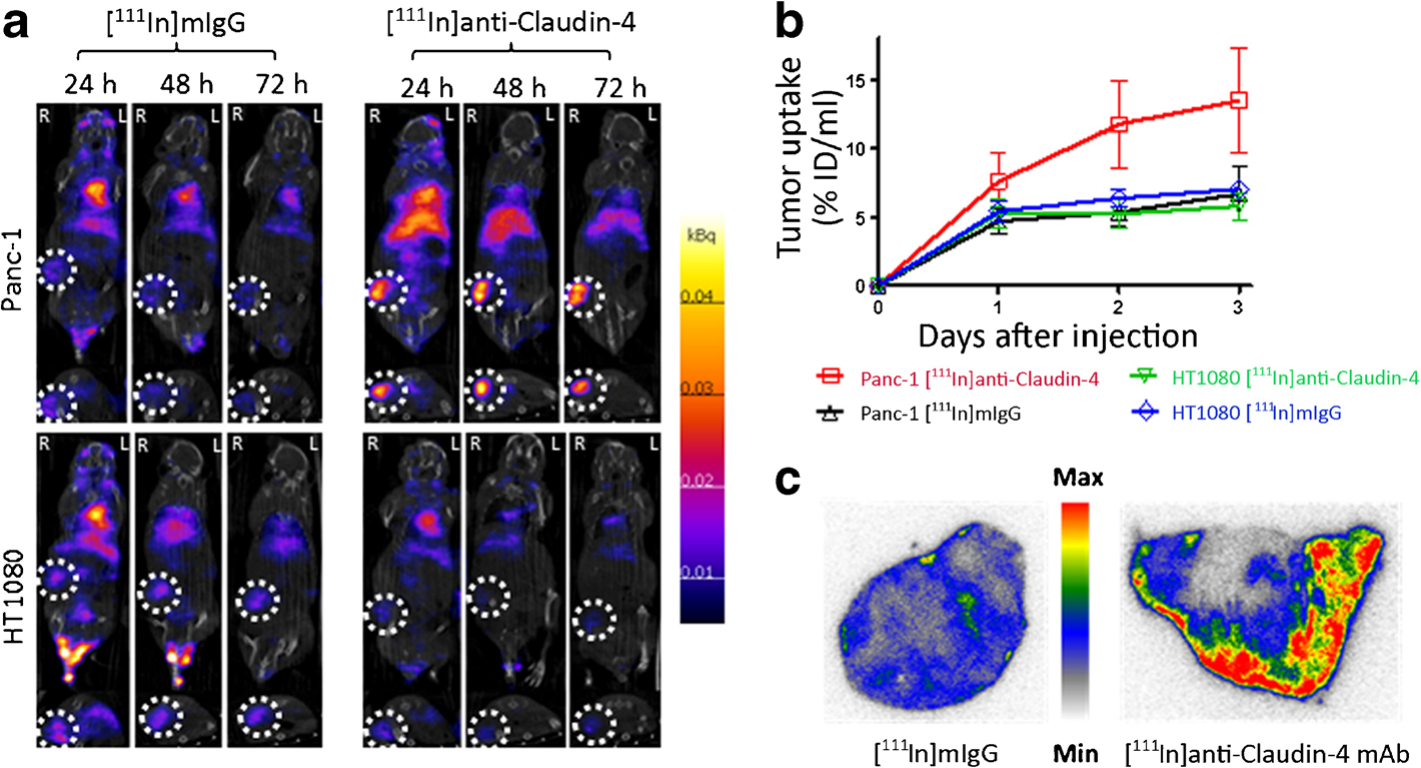
**a** Coronal and transaxial SPECT/CT images of mice bearing Panc-1 (top row) or HT1080 (bottom row) xenograft tumours. Coronal images depict ventral views of the mice. Mice were administered either [^111^In]anti-claudin-4 or [^111^In]mIg intravenously and SPECT/CT images were acquired at 24, 48 and 72 h post-injection. Section thickness = 0.8 mm. **b** Time-activity curves generated based on volume-of-interest analysis of SPECT images showing overall tumour uptake expressed as % ID/ml ± SEM. **c** Autoradiography images of Panc-1 tumour xenograft sections revealing significantly higher uptake and penetration of [^111^In]anti-claudin-4 mAb compared to [^111^In]mIgG at 3 days p.i.

Representative SPECT/CT images obtained at 24, 48 and 72 h post-injection of [^111^In]anti-claudin-4 mAb or [^111^In]mIgG (an isotype-matched control antibody lacking claudin-4 specificity) are shown in Fig. 3a. Volume-of-interest analysis revealed that uptake of [^111^In]anti-claudin-4 within claudin-4-overexpressing Panc-1 tumours increased over time, yielding uptake values of 9.5 ± 1.9, 14.7 ± 3.0 and 16.9 ± 4.5 % ID/ml at 24, 48 and 72 h post-injection (p.i.), respectively (Fig. 3b). Tumour uptake of [^111^In]anti-claudin-4 in Panc-1 xenografts was significantly higher compared to that obtained with [^111^In]mIgG at all time points (*P* < 0.01) and with [^111^In]anti-claudin-4 in HT1080 tumours (*P* < 0.01). Furthermore, uptake of [^111^In]anti-claudin-4 in HT1080 (no claudin-4 expressing) tumour xenografts was identical to that of [^111^In]mIgG and therefore did not exceed the level of non-specific uptake resulting from the enhanced permeability and retention effect [17]. Taken together, these measurements strongly indicate that uptake of [^111^In]anti-claudin-4 in Panc-1 tumour xenografts was primarily mediated by claudin-4 expression.

The overall *in vivo* biodistribution of [^111^In]anti-claudin-4 is typical for a radiolabelled whole immunoglobulin. Due to its long residence time within the circulation and excretion *via* the hepatobiliary system, high signals can also be observed within the blood pool (heart and carotid arteries) and liver.

At 72 h p.i., the *ex vivo* biodistribution data (Table 1) was consistent with the data extracted from the SPECT images. Overall uptake of [^111^In]anti-claudin-4 was significantly higher in Panc-1 compared with HT1080 tumours (14.3 ± 2.6 and 4.3 ± 3.0 % ID/g, respectively; *P* < 0.001) and was also higher compared to uptake of [^111^In]mIgG in Panc-1 (6.3 ± 0.8 % ID/g; *P* < 0.01) and HT1080 (5.4 ± 0.9 % ID/g; *P* < 0.01) tumours. The tumour-to-blood ratio (T/B) obtained with [^111^In]anti-claudin-4 at 72 h p.i. was also significantly higher for Panc-1 compared with HT1080 tumour-bearing mice (1.3 ± 0.1 and 0.4 ± 0.2, respectively; *P* < 0.001) and was higher compared to the T/B values obtained with [^111^In]mIgG in Panc-1 (0.6 ± 0.0; *P* < 0.001) and HT1080 (0.6 ± 0.2; *P* < 0.001) tumour-bearing mice. Similar conclusions were drawn for tumour-to-pancreas and tumour-to-intestines ratios, as derived from Table 1.

**Table 1:** *Ex vivo* biodistribution data acquired at 72 h p.i. of [^111^In]anti-claudin-4 mAb or [^111^In]mIgG

	[^111^In]anti-claudin-4 Panc-1 (*n* = 5)	[^111^In]mIgG Panc-1 (*n* = 3)	[^111^In]anti-claudin-4 HT1080 (*n* = 5)	[^111^In]mIgG HT1080 (*n* = 3)
Blood	10.61 ± 0.78	9.91 ± 1.73	10.41 ± 2.15	8.81 ± 3.24
Tumour	14.31 ± 2.60	6.26 ± 0.82	4.27 ± 3.01	5.38 ± 0.90
Heart	2.84 ± 0.47	3.71 ± 0.49	2.19 ± 0.90	2.93 ± 1.17
Lung	4.27 ± 0.44	5.13 ± 0.15	1.79 ± 1.23	4.14 ± 1.65
Liver	4.50 ± 0.59	4.13 ± 0.40	3.55 ± 0.48	4.47 ± 1.33
Spleen	5.92 ± 0.78	5.50 ± 1.50	3.19 ± 2.23	5.87 ± 1.81
Stomach	0.75 ± 0.32	0.64 ± 0.30	0.50 ± 0.14	0.37 ± 0.11
Large intestine	1.03 ± 0.19	1.20 ± 0.12	0.95 ± 0.24	0.80 ± 0.27
Small intestine	1.37 ± 0.28	1.40 ± 0.21	0.71 ± 0.27	1.23 ± 0.26
Pancreas	1.95 ± 0.40	1.96 ± 0.26	1.04 ± 0.43	1.47 ± 0.57
Kidney	4.56 ± 0.60	5.21 ± 0.28	3.34 ± 0.75	4.04 ± 0.73
Muscle	1.14 ± 0.31	1.12 ± 0.31	0.57 ± 0.23	0.83 ± 0.17
Skin	3.18 ± 0.74	3.03 ± 1.26	1.32 ± 0.85	2.04 ± 0.51
Fat	3.09 ± 1.34	2.24 ± 0.29	2.18 ± 1.08	3.50 ± 2.07

Uptake values are reported as % ID/g ± SD

Autoradiography of frozen xenograft sections (Fig. 3c) revealed higher accumulation of [^111^In]anti-claudin-4 within Panc-1 tumours compared with [^111^In]mIgG. Furthermore, [^111^In]anti-claudin-4 showed that moderate penetrative ability as radioactivity can be observed extending diffusely into the core of the tumours.

### Imaging of Claudin-4 in KPC Mice

KPC mice showing a positive indication of PDAC by [^18^F]FDG-PET and MRI screening were injected with [^111^In]anti-claudin-4 or non-specific [^111^In]mIgG and imaged by SPECT. Pancreatic uptake of [^111^In]anti-claudin-4 in KPC mice at 72 h post-injection as measured by *ex vivo* gamma-counting was found to be slightly higher when compared to that of [^111^In]mIgG (3.14 ± 0.81 *vs.* 2.58 ± 0.80 % ID/g, see Table S1). However, pancreas-to-blood ratios were found to be similar for both tracers (~ 0.35), suggesting that [^111^In]anti-claudin-4 was unable to provide enough contrast to delineate claudin-4 overexpression in pancreatic tissue, when the whole pancreas was measured.

**Fig. 4.**
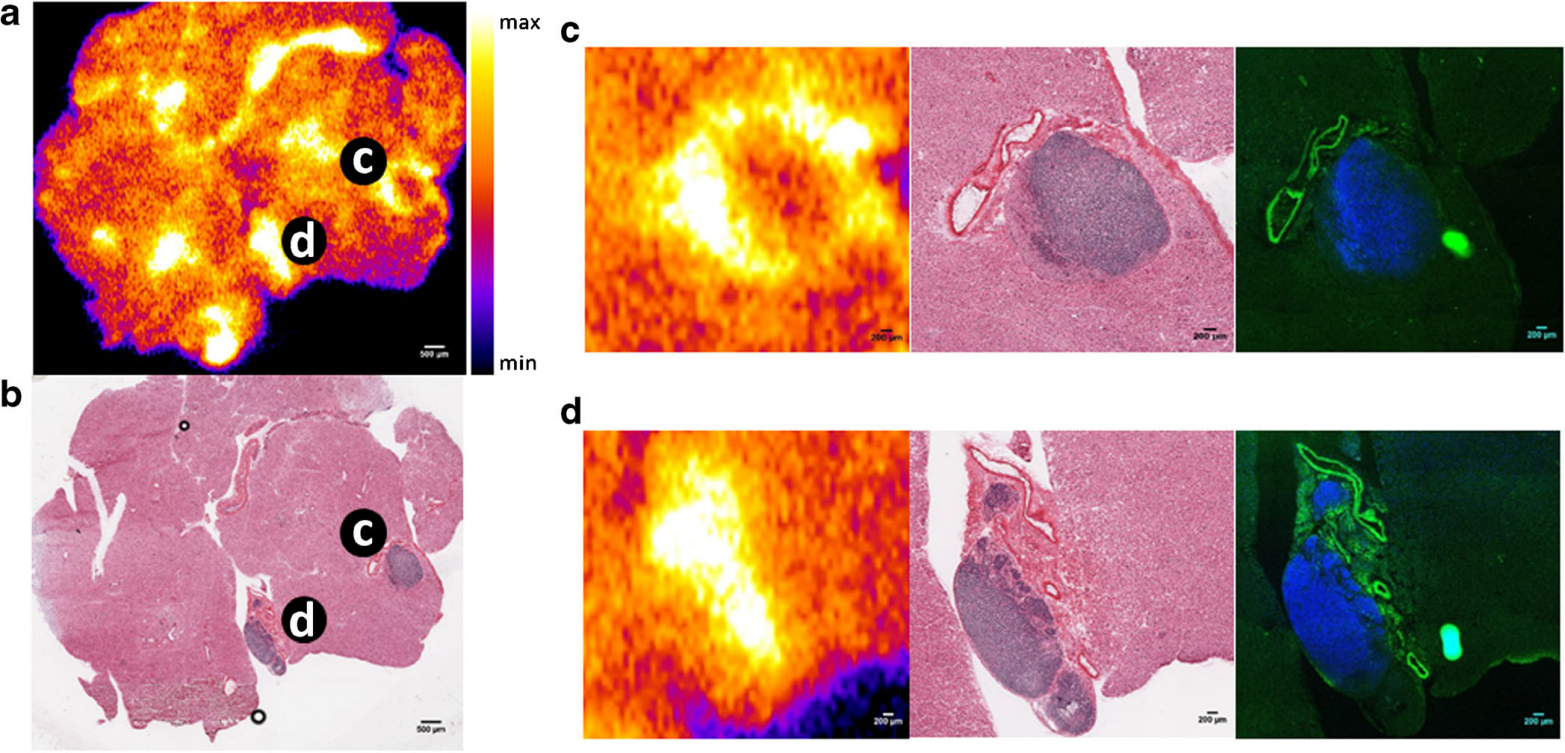
**a** Autoradiograph of pancreatic tissue section from KPC mouse injected with [^111^In]anti-claudin-4 and **b** corresponding H&E photomicrograph. **c**, **d** Magnified views of representative high-uptake regions. Visual inspection of *ex vivo* autoradiographs revealed a number of distinct [^111^In]anti-claudin-4 hotspots within tissue, while [^111^In]mIgG exhibited a more diffuse pattern of distribution (Fig. S2, supplementary information). Co-registration of autoradiography, H&E and immunofluorescence images demonstrated that [^111^In]anti-claudin-4 hotspots coincided with areas showing signs of PDAC pathology and claudin-4 expression, as shown by H&E staining and immunofluorescence (blue: DAPI, green: claudin-4).

Autoradiography of KPC pancreas sections revealed that a heterogeneous, focal distribution of [^111^In]anti-claudin-4 within tissue while [^111^In]mIgG exhibited a more diffuse pattern of accumulation (Fig. 4 and S2, respectively). The presence of claudin-4 and PDAC lesions in the same tissue sections was confirmed by immunofluorescence and H&E staining, respectively. Interestingly, co-registration of autoradiography, immunofluorescence and H&E images showed a clear association between [^111^In]anti-claudin-4 uptake and claudin-4 expression in pancreatic ducts affected by PDAC (see Fig. 4).

## Discussion

The progression of early preneoplastic PanIn lesions to invasive PDAC in humans is known to occur over several years, which provides a broad window of opportunity to diagnose this malignancy when interventional therapy is most likely to succeed [18]. The development of non-invasive imaging methods capable of detecting biomarkers associated with high-grade PanIn lesions would aid in the identification of patients most at risk of developing PDAC. In recent years, several biomarkers, signalling pathways and gene aberrations of PDAC have been discovered and are frequently the basis of novel targeted therapies in preclinical studies [8, 9, 19, 20]. However, examples of imaging agents directed against these early indications of pancreatic cancer are comparatively rare.

Considerable efforts have been made to understand the role of claudin-4 in the development of PDAC and a variety of other cancer types. Gene expression analyses have revealed that claudin-4 is increasingly expressed in pancreatic cancer as it progresses into more advanced stages [21, 22]. Interestingly, several studies have demonstrated that claudin-4 protein expression is associated with decreased invasiveness and reduced metastatic potential, and it has been positively correlated with better prognosis in PDAC [6, 23]. Similar conclusions have been drawn regarding its involvement in colorectal cancer [24], oesophageal squamous cell carcinoma [25] and gastric cancer [26–28]. Conversely, claudin-4 overexpression has been positively correlated with increased invasiveness [29–31], metastasis [32, 33], angiogenesis [34] and poor prognosis [35, 36] in several other cancer types, including breast [32, 35, 37, 38], gastric [31, 36, 39], lung [40], ovarian [29, 34, 41, 42], prostate [33] and uterine cancers [43]. Taken together, these results highlight the utility of claudin-4 overexpression not only as an early detection marker for many different cancers, but also as a cancer type-specific prognostic indicator.

Recent efforts to develop molecular imaging agents for the detection of claudin-4 have relied on the use of CPE fragments as targeting vectors [12, 13, 44]. While these approaches have shown great potential for delineating claudin-4 expression in tumour tissue and precancerous lesions *in vivo*, CPE-based imaging agents suffer from poor solubility, unknown immunogenicity and exhibit only moderate affinity and specificity for claudin-4.

Despite their slower kinetics, antibodies are considered an attractive alternative to CPE for molecular imaging of claudin-4 due to their superior target affinity and specificity. Foss and co-workers first reported specific binding of an iodine-125 radiolabelled anti-claudin-4 mAb to claudin-4 in subcutaneous and orthotopic xenograft models of PDAC *in vivo* by SPECT imaging [45]. However, the conspicuously low level of radioactivity in the blood (1.21 ± 0.77 % ID/g) at 48 h p.i. is atypical of an antibody and is suggestive of rapid dehalogenation which is a common issue with radioiodinated antibodies.

In the present study, we developed a refined antibody-based SPECT imaging agent for the delineation of claudin-4 expression *in vivo*. We selected a human anti-claudin-4 monoclonal antibody as a suitable targeting vector on the basis of its high binding affinity and specificity for human and murine claudin-4. Unlike the probe developed by Foss et al., this antibody was specifically modified with a bifunctional *p*-SCN-Bn-DTPA chelator in order to enhance the stability of the radioimmunoconjugate. Modification of the chosen antibody with *p*-SCN-Bn-DTPA led to a minimal reduction in binding affinity for the target protein. Although indium-111 was chosen as a radiolabel in this study due to its long half-life and ready availability, this probe could also be adapted to PET imaging in order to facilitate quantitative image analysis in the clinical setting.

[^111^In]anti-claudin-4 mAb revealed a promising ability to bind to its target antigen in human xenograft models of pancreatic cancer. Total uptake of [^111^In]anti-claudin-4 mAb in Panc-1 tumours at 72 h p.i. was approximately 3-fold higher compared with experimental controls. Tumour uptake of [^111^In]anti-claudin-4 mAb in Panc-1 xenografts at 72 h p.i. was markedly higher than the maximum value reported by Foss and co-workers in the same model (4 % ID/g at 48 h p.i.), probably due to the superior kinetic stability of the radioimmunoconjugate [45].

Conversely, no significant differences in total pancreatic uptake between [^111^In]anti-claudin-4 mAb and [^111^In]mIgG were detected in the KPC mouse model. This could be due to a more heterogeneous pattern of claudin-4 expression in the KPC pancreas compared to human pancreatic cancer xenografts as a result of the presence of different grade PDAC lesions within tissue, as suggested by our immunofluorescence results. Encouragingly, co-registration of autoradiography and histology images of pancreatic tissue sections from KPC mice indicated that the sites showing the most prominent [^111^In]anti-claudin-4 mAb accumulation coincided with ductal regions exhibiting clear signs of PDAC pathology and high claudin-4 expression levels. Nevertheless, the poor contrast provided by [^111^In]anti-claudin-4 mAb in the KPC model could also be the result of limited access to the tumour mass due to the presence of dense fibrous stroma, as well as non-specific accumulation of the radioimmunoconjugate in the liver and spleen. Further improvements in tumour-to-background ratios could be feasibly achieved by utilising a smaller antibody fragment which would undergo faster blood clearance and renal elimination. Pretargeted imaging strategies [46] may also offer an alternative solution, particularly as the high cell surface persistence of claudin-4 would render it a suitable candidate for this approach.

## Conclusions

[^111^In]anti-Claudin-4 mAb is a useful tool for the non-invasive SPECT imaging of claudin-4 which is a widely dysregulated and highly prognostic biomarker in pancreatic cancer. This imaging agent could therefore be used to aid in the early detection and characterisation of this malignancy.

## Electronic supplementary material

ESM 1 (PDF 314 kb)

## Abbreviations

PDAC: Pancreatic ductal adenocarcinoma
CT: Computed tomography
PET: Positron emission tomography
SPECT: Single-photon emission computed tomography
MRI: Magnetic resonance imaging
[^18^F]FDG: 2-deoxy-2-[^18^F]fluoro-D-glucose
PanIn: Pancreatic intraepithelial neoplasia
CPE: *Clostridium perfringens* enterotoxin
% ID/g: % of injected dose per gram
