# Supplementary material for: Imaging of Claudin-4 in Pancreatic Ductal Adenocarcinoma Using a Radiolabelled Anti-Claudin-4 Monoclonal Antibody
Source: Mol Imaging Biol. 2017 Aug 25;20(2):292–9. doi: 10.1007/s11307-017-1112-8 (PMC5862916; doi:10.1007/s11307-017-1112-8)
Supplement: Supplementary file 1 — (PDF 314 kb) [file 11307_2017_1112_MOESM1_ESM.pdf]

Electronic Supplementary Material

Imaging of Claudin-4 in Pancreatic Ductal Adenocarcinoma using a Radiolabelled Anti-Claudin-4 Monoclonal Antibody

Journal: Molecular Imaging and Biology

Julia Baguña Torres[1,*], James C. Knight[1,*], Michael J. Mosley[1,*], Veerle Kersemans[1], Sofia Koustoulidou[1], Danny Allen[1], Paul Kinchesh[1], Sean Smart[1], and Bart Cornelissen[1†]

[1] *CR-UK/MRC Oxford Institute for Radiation Oncology, Department of Oncology, University of Oxford, Oxford, United Kingdom*

† Corresponding author – email: bart.cornelissen@oncology.ox.ac.uk; address: Old Road Campus Research Building, Off Roosevelt Drive. Oxford OX3 7DQ; telephone: ++44 (0) 1865 857126

METHODS AND MATERIALS

Cell culture

Panc-1 (human pancreatic duct adenocarcinoma), HT1080 (human connective tissue epithelial fibrosarcoma) and 4T-1 (mouse mammary gland epithelial breast cancer cell) cell lines were obtained from the American Type Culture Collection (ATCC). Each of these cell lines was maintained in Dulbecco's Modified Eagle Medium (DMEM), supplemented with 10% fetal bovine serum (FBS), 2

mM L-glutamine, 100 units/ml penicillin, and 0.1 mg/ml streptomycin. All cell lines were cultured in a 37°C environment containing 5% CO_2. Cells were harvested and passaged as required using Trypsin-EDTA solution. Cells were tested and authenticated by the providers. The cumulative length of culture was less than 6 months following retrieval from liquid nitrogen storage.

Antibodies

Flow Cytometry

All procedures were performed on ice unless otherwise stated, and flow cytometry buffer (FC: 1% BSA, 1 mM EDTA, 0.1% sodium azide, 25 µg/ml DNASe I, 5 mM $MgCl_2$ in calcium/magnesium-free phosphate buffered saline [PBS]) was used throughout. Panc-1, HT1080 and 4T-1 cells were detached from culture flasks with Accutase EDTA, washed in PBS, resuspended in FC buffer, and counted. The cell suspension was made to 2.5×10^6 mL^{-1} and blocked in FC buffer for 20 min at room temperature. Cell suspensions (5×10^5 cells, 200 µl) were incubated with serial dilutions of either unmodified anti-claudin-4 mAb (MAB4219, R&D Systems) or anti-claudin-4 mAb modified with *p*-SCN-Bn-diethylenetriaminepentaacetic acid (DTPA) for 1 h at room temperature. Cells were then washed twice in FC buffer and incubated with a 1:300 dilution of a goat anti-mouse IgG Alexa Fluor® 488 fluorescently tagged secondary antibody (Life Technologies A11001) for 1 h at room temperature. After washing, the cells were resuspended in FC Buffer (500 µl) and analysed on a BD FACScan™ automated flow cytometer. Analysis of the data from 3 separate experiments was performed using the FlowJo Single Cell Analysis software (Treestar, San Carlos, CA, USA).

Western immunoblot

Total protein preparations were performed at 4°C on approximately 1×10^7 panc-1 or HT1080 cell lines using RIPA lysis buffer (50 mM Tris, pH 8, 1% NP40, 0.5% sodium deoxycholate, 0.1% sodium dodecyl sulphate, 150 mM sodium chloride, cOmplete™ protease inhibitor cocktail [Sigma-Aldrich]). The cell lysates were isolated by centrifugation after lysis through a 21G hypodermic syringe and 30 second sonication. Thirty microgram lysate samples were run on a 4-12% Bis-Tris MES gel (Novex), transferred to a PVDF membrane and exposed to a 1:700 dilution of anti-claudin-4 antibody (329400,

ThermoFisher Scientific), followed by a 1:3000 dilution of the secondary goat anti-mouse-HRP (5178-2504, Bio-Rad). The membrane was exposed to autoradiography film after development using an ECL western blot substrate solution (Pierce Thermo Scientific 32209).

Immunofluorescence

Frozen tumour xenograft and pancreatic tissue sections (10 µm) were allowed to reach room temperature, washed in PBS and fixed in 4% paraformaldehyde solution for 10 min. After washing, the sections were permeabilized in 0.5% digitonin for 20 min at room temperature, washed again and blocked in 2% BSA for 1 h at room temperature. The sections were then hybridized to a 1:100 dilution of anti-claudin-4 antibody (PA5-28830, ThermoFisher Scientific) at 4°C overnight, washed and incubated with a 1:500 dilution of a goat anti-rabbit IgG Alexa Fluor® 488 (Invitrogen A11034) for 1 hour at room temperature. After further washing, a small drop of Vectashield plus DAPI (Vector H-1200) was applied to each section and a coverslip placed on the slide and secured with Covergrip (Biotium 23005). The slides were viewed on a Leica 90i microscope (Leica Microsystems).

In Vivo

Xenograft study

SPECT/CT Imaging

SPECT/CT images were acquired using a 4-head multi-pinhole nanoSPECT-CT scanner (Bioscan, Washington, USA), calibrated by imaging a phantom with an indium-111 standard solution. Animals were anaesthetised by 4% isoflurane gas (0.5 l/min O_2) and maintained at 2% and 37°C throughout the imaging session. The temperature of the animals was maintained at 37°C, using a custom-built mouse cradle. Image acquisition was performed over 1 h in 24 projections, aiming for 100.000 counts/projection, using a 1.5 mm pinhole collimator. Whole-body CT images were acquired at a tube setting of 45 kVp, 177 µA, 500 ms per view, for anatomical reference. Reconstruction of both CT and SPECT images was performed with the HiSPECT algorithm using InVivoScope (Version 1.42,

Bioscan). VOI analyses were performed using the Inveon Research Workplace software package version 2.2 (Siemens Preclinical Solutions).

KPC study

The spontaneous development of PDAC in male KPC mice was assessed every 2 weeks by sequential acquisition of T2 weighted MRI and [^{18}F]FDG-PET imaging data. These scans occurred within a single anaesthetic session during which each mouse was immobilised and transferred between imaging systems within a custom-built cradle. This allowed reliable co-registration of data from both imaging systems. The high spatial resolution and excellent soft-tissue contrast provided by MRI enabled accurate measurements of tumour dimensions within the pancreas. The gradual formation of PDAC was reflected by gross anatomical changes which were clearly distinguished by MRI. In all cases where a positive indication of PDAC could be identified by MRI, [^{18}F]FDG uptake was also observed within the tumour. The animals found to be PDAC-positive by these methods were used for claudin-4 imaging studies.

<u>SPECT/PET/CT imaging</u>

SPECT/PET/CT images were acquired using a VECTor^{4}CT integrated PET/SPECT/CT system (MILabs, Utrecht, The Netherlands), which allows simultaneous PET and SPECT imaging. KPC mice were intravenously injected with [^{111}In]anti-claudin-4 or [^{111}In]mIgG (5 MBq, 5 µg) administration (n=3 per tracer) and imaged by SPECT 72 h after radiotracer administration. Prior to SPECT acquisition, animals were fasted for 4 - 8 h and then injected intravenously with [^{18}F]FDG (5 MBq). Water was provided ad libitum. After an [^{18}F]FDG uptake period of 1 h, mice were anaesthetised by 4% isoflurane gas (0.5 L/min O_2) and placed on a custom-built imaging cradle in a prone position. SPECT/PET acquisition (3 frames, 8 min 20 s per frame, 50 s per bed position) was performed for 25 min using an ultra-high resolution rat/mouse 1.8 mm collimator, followed by a cone-beam CT scan (55 kV, 0.19 mA) for anatomical reference and attenuation correction. Anaesthesia was maintained at 2.5 % isoflurane throughout the duration of the scans.

SPECT and PET images were reconstructed using U-SPECT-Rec3.22 software (MILabs, Utrecht, The Netherlands), applying a pixel-based algorithm with 8 subsets, 6 iterations and 0.8 mm voxel size for both ^{111}In (161.1-196.9 and 227.7-278.3 keV) and ^{18}F (477.9-584.1 keV).

MRI imaging

Immediately following SPECT/PET/CT imaging and during the same anaesthetic session, mice were transferred within the same cradle to the MRI scanner. MRI was performed at 7.0 T (VNMRS, Varian Inc., Palo Alto, CA) using a 32 mm ID quadrature birdcage coil (Rapid Biomedical GmbH, Rimpar, Germany). A respiratory gated 3D balanced SSFP (bSSFP) sequence[1] with TR 3.268 ms, TE 1.134 ms, FOV 64×32×32 mm^3, matrix 256×128×128 and a 15° degree flip with a 16 μs hard pulse.[2-3] Scans were performed with and without RF phase alternation in order to allow the production of a maximum-intensity projection image that features reduced susceptibility artefact. Additionally, a respiratory gated 2D multi echo CPMG sequence using SPLICER[4] with TR 4.38 s, 8 echoes with first TE and echo spacing 6.856 ms, FOV 64×32 mm^2, matrix 192×96, 72 contiguous 0.33 mm thick slices, was used to aid tumour identification. The MR image was registered to the CT image using the rigid body registration 'imregister' function built into MATLAB. A further non-rigid registration step was required after to compensate for the non-linear spatial distortions inherent in MR imaging, and was performed using the 'MIND' algorithm[5].

Ex vivo

H&E staining

After autoradiography, tissue sections were washed under running water for 5 min and subsequently stained with filtered Mayer's haematoxylin (Sigma-Aldrich) for 2 min. Slices were then rinsed in dH_2O for 20 min prior to staining with 1% eosin (v/v) for 10 s. Finally, sections were dehydrated in increasing concentrations of ethanol (70%, 95%, and 100% (v/v) in dH_2O; Fisher Scientific) and cleared in xylene (Fisher Scientific). Slides were mounted using DPX mounting medium (Fisher Scientific) and scanned using a ScanScope CS system (Aperio, USA).

RESULTS

Figure S1: Determination of MAB4219 affinity for murine claudin-4 in 4T-1 cells by flow cytometry. The saturation signal was lower compared to that obtained for the Panc-1 cell line but considerably higher than that measured in claudin-4-negative HT1080 cells. The K_d value of the interaction was 108±22 nM.

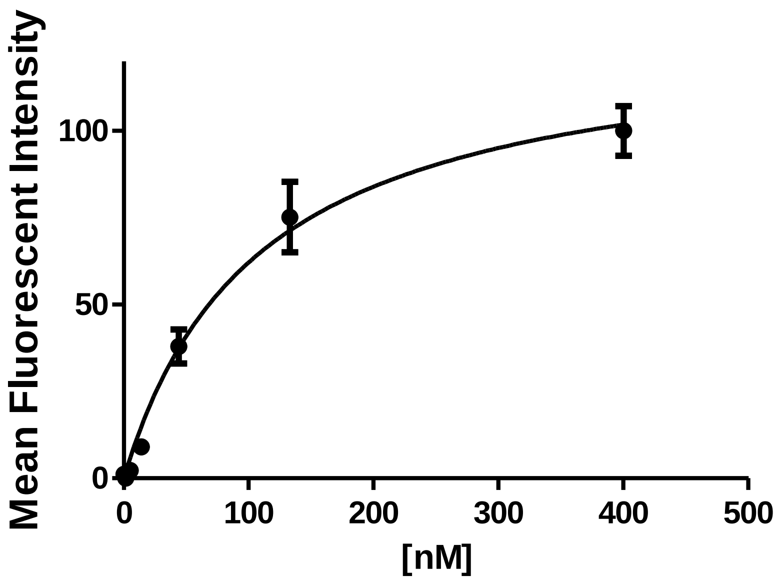

Table S1: *Ex vivo* biodistribution data acquired at 72 h p.i. of [^{111}In]anti-Claudin-4 or [^{111}In]mIgG in KPC mice. Values are % ID/g ± SD (n=3 per tracer).

	**[^{111}In]anti-Claudin-4**	**[^{111}In]mIgG**
Blood	9.00±1.45	7.37±1.33
Heart	3.09±0.19	2.06±0.61
Lung	4.56±0.47	3.23±0.59
Liver	5.14±0.98	4.21±0.96
Spleen	5.52±0.30	4.50±2.10
Stomach	2.87±0.56	2.23±1.11
Large intestine	2.65±1.25	2.78±0.88
Small intestine	1.75±0.45	1.50±0.85
Pancreas	3.14±0.81	2.58±0.80
Kidney	5.54±1.99	4.28±3.69
Muscle	0.87±0.34	0.96±0.58
Skin	2.63±1.08	1.59±0.42
Fat	1.94±1.75	2.59±0.89

Figure S2: Autoradiograph of pancreatic tissue section from KPC mouse injected with [^{111}In]mIgG and corresponding H&E photomicrograph. No correlation was found between [^{111}In]mIgG uptake in pancreatic tissue and PDAC pathology, as depicted by H&E staining. Co-registration of autoradiography, H&E and immunofluorescence images showed that [^{111}In]mIgG uptake was not specific to regions with high expression of claudin-4.

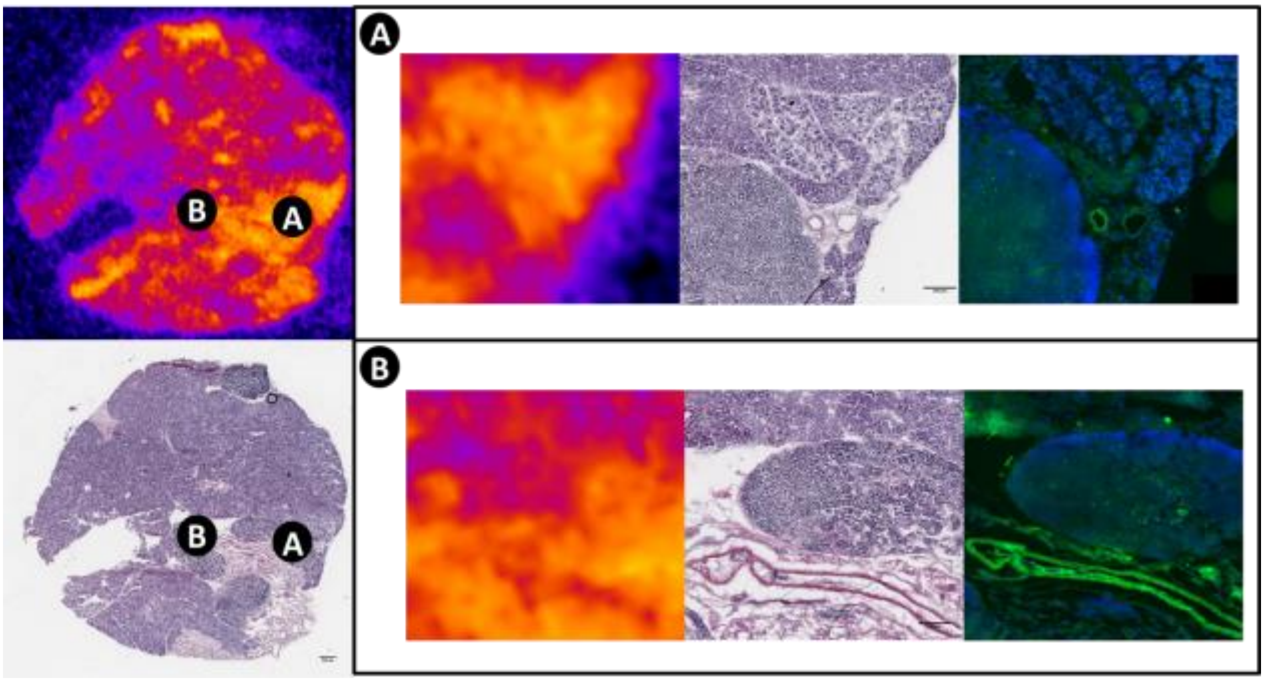

References

1. Oppelt A, Graumann R, Barfuss H, Fischer H, Hartl W, Schajor W (1986) FISP, a novel, fast pulse sequence for nuclear magnetic resonance imaging. Electromedica 54:15-18.
2. Scheffler K, Lehnhardt S (2003) Principles and applications of balanced SSFP techniques. European Radiology 13:2409-2418.
3. Kinchesh P, Gilchrist S, Gomes AL, et al. (2016) Accelerated Imaging of the Mouse Body using k-space Segmentation, Cardio-Respiratory

Synchronisation and Short, Constant TR: Application to b-SSFP. In ISMRM 24th Annual Meeting and Exhibition. Singapore.
4. Kinchesh P, Allen PD, Beech JS, et al. (2015) Dynamic Reacquisition for Respiratory Gated, Constant TR 2D multi-slice MRI. In ISMRM 23rd Annual Meeting and Exhibition. Toronto, ON, Canada.
5. Heinrich MP, Jenkinson M, Bhushan M, et al. (2012) MIND: Modality independent neighbourhood descriptor for multi-modal deformable registration. Medical Image Analysis 16:1423-1435.
